# Ethnoveterinary medicinal plants: Preparation and application methods by traditional healers in selected districts of southern Ethiopia

**DOI:** 10.14202/vetworld.2015.674-684

**Published:** 2015-05-29

**Authors:** Gebremedhin Romha Eshetu, Tewedros Ayalew Dejene, Lidet Befkadu Telila, Daniel Fekadu Bekele

**Affiliations:** 1 Department of Animal and Range Science, College of Agriculture and Natural Resource, Dilla University, P.O. Box 419, Dilla, Ethiopia; 2 Department of Plant Science, College of Agriculture and Natural Resource, Dilla University, P.O. Box 419, Dilla, Ethiopia; 3 Department of Horticulture, College of Agriculture and Natural Resource, Dilla University, P.O. Box 419, Dilla, Ethiopia; 4 Institute of Indigenous Studies, Dilla University, P.O. Box 419, Dilla, Ethiopia

**Keywords:** animal ailments, ethnoveterinary medicinal plants, indigenous knowledge, traditional healers

## Abstract

**Aim:**

The aim was to document the ethnoveterinary medicinal plants, their preparation, and application methods used by traditional healers in treating different animal diseases, in four districts with different culture and languages in southern Ethiopia.

**Materials and Methods:**

Information of ethnoveterinary medicinal plants was obtained through in-depth direct interview with the local healers and field observations. A descriptive statistics was used to analyze the reported ethnoveterinary medicinal plants and associated indigenous knowledge. The informant consensus factor (ICF) was calculated for each category of diseases to identify the agreements of the informants on the reported cures. Preference ranking was used to assess the degree of effectiveness of certain medicinal plants against most prevalent animal diseases in the area.

**Results:**

The healers had a very high intention to keep their traditional knowledge secrete and none of them was ready to transfer their knowledge either freely or on incentive bases to other people; they need to convey their knowledge only to their selected scions after getting very old. A total of 49 plant species used to treat 26 animal ailments were botanically classified and distributed into 34 families. The most commonly used plant parts for remedy preparations were leaves (38.8%), followed by whole roots (20.4%). *Calpurnia aurea* (Ait.) Benth was the most preferred effective treatment against external parasite and skin problem, which is the most prevalent disease with the highest ICF (0.68).

**Conclusion:**

The study suggests that the community of the study districts depend largely on ethnoveterinary medicinal plants for the treatment of different animal ailments though the healers have a very high intention to keep their traditional knowledge secrete. Commonly reported plant species need to be tested for their antimicrobial activities *in vitro* and validated their active ingredients in order to recommend effective preparations and treatments to this community.

## Introduction

Enthnoveterinary medicine is a holistic interdisciplinary study of the local knowledge and the socio-cultural structures and environment associated with animal health care and husbandry [1]. Hence, to keep animals healthy, traditional healing practices have been applied for centuries and have been passed down orally from generation to generation [2,3]. Widespread interest in documenting and validating ethnoveterinary practices arose in the early 1980s. Since then, several studies have been carried out, many reports written, and numerous conferences and workshops held. These activities have saved ethnoveterinary knowledge from extinction because most knowledge resided with elderly community members and disappeared as they died [2,3]. However, the effort is still quite insignificant when compared to the undocumented global ethnoveterinary plant lore.

In Ethiopia, animal disease remains one of the principal causes of poor livestock performance, leading to an ever increasing gap between the supply of, and the demand for livestock products [4]. Conventional veterinary services, despite its paramount role, have limited coverage in developing countries [5,6]. Due to this reason livestock keepers particularly in rural areas frequently visit traditional healers to get solutions for their ill-health animals; they complement modern medicine by developing a socially acceptable remedy from inexpensive resources.

The traditional knowledge on ethnoveterinary practices by local healers who are knowledgeable and experienced in traditional systems of treatment is important, but their knowledge are not documented and is dwindling fast [7]. It is also indicated that the knowledge of ethnomedicinal plants is on the verge of irreversible loss and declining to deterioration due to the oral passage of herbal heritage from generation to generation rather than in writings, despite their vital role in catering for the health of human, and livestock population [8]. Environmental degradation, agricultural expansions, cultivation of marginal lands, and urbanization are also posing a significant threat to the future wellbeing of human and animal populations that have relied on these resources to combat various ailments for generations [9-11] warranting urgent need to document and preserve the indigenous knowledge. Hence, it is a timely endeavor to document, promote and conserve the country’s ethnoveterinary medicinal plant lore. Such documents are important to define and maintain cultural identity of the people [12] in addition to serving as keys toward establishing people-centered natural resource management systems [13], and potentials for scientific discovery of new compounds used in the development of modern drugs [14].

Although attempts have been made to document Ethiopian ethnoveterinary medicinal plants in some cultural groups [8,15-20], it is found insignificant when compared to the multi-ethnolinguistic communities found in the country, which have remained largely unexplored. Therefore, the present study was designed to document the ethnoveterinary medicinal plants, and their preparation and application methods used by traditional healers in treating different animal diseases, in the four districts with different culture and languages; Yabelo and Liben districts in Oromia region, and Wondo Genet and Kochere districts in southern nations region, southern Ethiopia.

## Materials and Methods

### Ethical approval

This study was approved by the Research and Dissemination Committee of Dilla University. The confidentiality of traditional property owners was completely maintained during processing our data. In addition, all data from this study were not shared with third party out of researchers. Informed consent was also obtained from the participants to ensure their willingness.

### Description of the study areas

**Figure-1:**
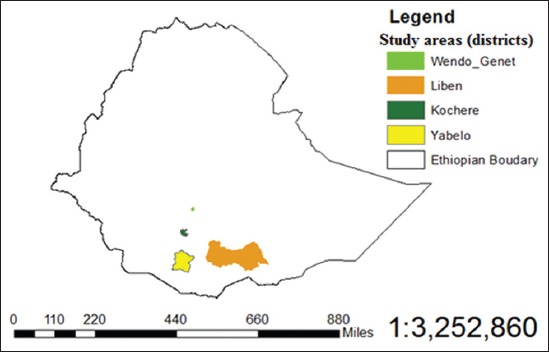
Location of the study districts.

This study was conducted from May 2013 to July 2014 in the southern Ethiopia; Yabelo and Liben districts in Oromia regional state, Wondo Genet and Kochere districts in Southern Nations and Nationalities People Region (SNNPR) (Figure-1).

Liben and Yabelo districts are found in the Guji and Borena zonal administrative divisions of the Oromia Regional State. The people in the rural areas of both districts are pastoralists who make their livelihoods largely from livestock. They tend mixed herds of cattle, small ruminants, donkeys, and camels through seasonal movements between the wet and dry seasons grazing areas. Most of the areas in these districts are classified as lowlands, with ponds and wells serving as water sources for both people and livestock during the dry months [21]. The livestock population in Yabelo district is 1,496,652 cattle, 625,198 small ruminants, 75,644 equines, and 106,366 camels. Similarly, the livestock population in Liben district is 1,619,911 cattle, 1,003,027 small ruminants, 192,784 equines, and 235,105 camels (Borena and Guji zones pastoral development office, unpublished data).

The climatic condition in both districts is semi-arid with highly variable rainfall between 300 and 900 millimeters (mm) a year, with high spatial and temporal variability. Liben district is located 569 km south of the capital city of Ethiopia, Addis Ababa at about 310 km southeast of Hawassa. The altitude range of the area is between 1,000 m and 2,000 m above sea level (a.s.l.) with the coordinates 4° 40′-5° 20′N and 39° 10′-3° 30′E. Moreover, the town of Yabello is geographically found at 5° 23′49″N 39° 3′52″E, and located at a distance of 565 km Southern of Addis Ababa with an altitude 1000-1650 m a.s.l.

Wondo Genet, is found in Sidama Zone, SNNPR State, located on the western escarpment of the central rift valley of Ethiopia, that extend between 7° 06′N and 38° 37′E, 1720-2620 m a.s.l., about 272 km south of the capital city, Addis Ababa and about 24 km east of Hawassa Town. The Farming system practicing in the district is crop-livestock mixed farming. The dominant livestock being reared in the district is cattle (31,156), small ruminants (13,380), and equines (2,749). The climatic condition is humid and sub-humid with average annual temperatures of between 14°C and 20°C and annual rainfall between 700 mm and 1100 mm (Wondo Genet Agricultural and Rural Development Office, unpublished data).

Kochere district is geographically situated at 05° 55′-06° 07′N and 038° 15′-038° 5′E. It is at a distance of 420 km south of the capital city, Addis Ababa; 150 km south of the regional town Hawassa and 67 km east of the zonal town Dilla with an altitude ranging from 1500 to 3700 m a.s.l. The climatic condition of the district is humid with an average annual temperature and rainfall, 13-24°C and, 760-1500 mm, respectively. Crop-livestock mixed farming system is being practiced in Kochere district and the dominant livestock raised in the district is cattle (12,663), small ruminants (20,308), and equines (3,625) (data collection and dissemination work process of Gedeo Zone, unpublished data).

### Study design and selection of participants

A cross-sectional study was conducted using semi-structured questionnaires to gather information on the traditional usage of plants in the health care system of animals. The study districts were purposefully selected as these communities highly rely on traditional healings and possess many skills acquired from fore parents. Selection of informants was performed as in the manner described by Martin [22] who stated that when recording indigenous knowledge controlled by ethnobotanical healers or by certain social groups, the choice of key informant is vital. Thirty-one traditional healers (27 males and 4 females) were selected purposively based on the recommendation from local elders and governmental bodies; 13 from Liben and Yabelo districts (Oromia region), 13 from Wondo Genet district (Sidama zone), and 5 from Kochere districts (Gedeo zone) of southern nations region. The selected healers were well-known in the community due to their long practice in providing services related to traditional veterinary medicinal plants. The ages of the healers were between 35 and 91 years. During data collection, preliminary discussion was held with the individual key informants through assistance of the local elders and governmental bodies to elaborate the objective of the study.

### Plant specimen collections and identifications

Information of medicinal plants was obtained through a direct interview with the local healers and field observations. In-depth interview was also done to address details on the types and characteristics of plant and their traditional preparation, parts used, route of administration, dose given by the local healers, duration of the treatment, and other plants used together. Moreover, information was collected about the way of passing the indigenous knowledge from generation to generation. Voucher specimens were collected, coded by their vernacular names, pressed, and dried for identification. The plants identification was performed both in Dilla University in the department of biology by botany specialists [23,24], and at the National Herbarium of Addis Ababa University. All voucher specimens of the ethnoveterinary medicinal plants were labeled with scientific and vernacular names and stored in mini-herbarium in the College of Agriculture and Natural Resources, Dilla University.

### Quality assurance

To maintain the quality of data during interview, each informant was contacted at least 2 times for the same ideas and the validity of the information was proved and recorded. In case, the idea of the informant deviated from the original information, it was rejected as it was regarded irrelevant information. Only the relevant data were taken into account and statistically analyzed. Furthermore, the data quality was ensured through training of data collectors, checking of missing data, data cleaning, and careful data analysis.

### Data analysis

A descriptive statistical methods, percentage, and frequency were used to analyze the reported ethnoveterinary medicinal plants’ data and associated indigenous knowledge. The informant consensus factor (ICF) was computed for each category of diseases to identify the agreements of the informants on the reported cures for the group of diseases. The ICF was calculated as follows: Number of use citations in each category (nur) minus the number of species used (nt), and divided by the numbers of use citations in each category minus one [25].

Preference ranking was computed to assess the degree of effectiveness of certain medicinal plants against most prevalent diseases in the area. The medicinal plant that was believed to be the most effective was given the highest value, i.e., 5, and the one with the least effectiveness received the lowest value, i.e., 1 [22].


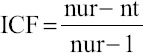


## Results

### Knowledge and practice toward ethnoveterinary medicinal plants

Of the 31 traditional healers interviewed, 29 (93.5%) reported that they acquired their knowledge from their parents or close relatives while 2 (6.5%) of the traditional healers responded that they acquired the medicinal plant knowledge on payment. The healers had a very high intention to keep their traditional knowledge secrete and none of them was ready to transfer their knowledge either freely or on incentive bases to other people. They need to convey their knowledge only to their selected scions (one herbalist can transfer his/her knowledge only to one of his/her selected son or daughter) after getting very old. According to the traditional healers, the medicine does not work if it is being told freely and sold to others.

### Diversity of ethnoveterinary medicinal plants

A total of 63 different ethnoveterinary medicinal plants used by various farmers of the study areas to treat a wide range of animal disease situations were collected and submitted to the National Herbarium of Addis Ababa University for botanical classification. Of these, 49 plant species having ethnoveterinary medicinal value were botanically classified and grouped under 34 families. About 24% of the botanical families were represented by more than one species. In this study, the highest number of plant species having ethnoveterinary medicinal value were found in *Euphorbiaceae, Fabaceae* and *Solanaceae*, with 4 plant species each followed by *Amaryllidaceae* (3 species). Four of the reported botanical families, i.e., Asteraceae, Cucurbitaceae, Meliaceae and Myrtaceae were represented by 2 species each. The remaining 26 (76%) families had a single species representation. A summary of the local and botanical names of the reported ethnoveterinary medicinal plants, and their indications, parts used, dose used by the local healers, preparations, and routes of administrations is presented in Table-1. Herbs were the most harvested for ethnoveterinary medicinal purpose and were represented with 18 (36.7%) plant species followed by 17 (34.7%) trees. Shrubs and climbers representing 7 (14.3%) plant species each (Table-2).

**Table-1:** Plant species identified as ethnoveterinary medicinal plants, and their preparation and application methods by the traditional healers in animals, in Wondo Genet, Yabelo, Liben and Kochere districts, southern Ethiopia.

Scientific name (family)	Family	Local name	Habit	parts used, preparation, route of administration, dosage, and duration	Disease treated	Species of animal	Voucher number
*Acmella caulirhiza* Del.	Asteraceae	Hajilo^d^	H	The fresh vegetative part of *Basella alba* L. and the flower of *Acmella caulirhiza* Del. are pounded, mixed together and squeezed, given 3 times/day (tid) until recovering:		Cattle, sheep, goat, and equine	AN29
				Orally	Bloat		
				Through eye	Eye problem		
				Topically	Wound		
*Albezia anthelimentica*	Fabaceae	Hawaachoo^b^	T	Chew up the fresh bark of the root of the plant by the local healer and then spit to the mouth of the animal about 1 teaspoonful, every day for 2 days	Internal parasite	Ruminants except pregnant	AN47
*Albizia schimperiana* Oliv.	Fabaceae	Gorbe^c^	T	The outer part of the fresh bark is removed and pounded then add about 200 g to 1 L of water and given 1 L (adult) and ½ L (young) orally 4 days interval until the disease ceases	Constipation	Cattle	AN35
*Albuca* spp.	Amaryllidaceae	Rada Waqa^b^	H	A fresh bulb (root) of the plant is ground and squeezed then 1 teaspoonful squeezed liquid added to 1 cup of water and is given 2 cup of the preparation through nose morning and evening for 4 days	Internal parasite	Cattle	AN20
*Albuca abyssinica* Jacq	Amaryllidaceae	Bute warbisa^b^	H	About 4 or 5 *Lapeirousia schimperi* and *Albuca* spp. piece fresh bulbs (roots) from each plant is pounded then add to 1 cup of water and given the victim immediately orally once	Snake bite	Ruminants Equine	AN8
*Allium sativum* L.	Amaryllidaceae	Qullubbii adii^b^	H	After pounding the bulb, add water, and filter then give through mouth and nose	Mastitis, diarrhea, internal parasite, and others	Cattle, sheep, goat, and equine	AN62
*Aloe scundiflora* Berger	Aloaceae	Hargeessa^b^	H	The sap of the plant is applied into the eye for 3 days	Eye disease	Ruminants chicken	AN51
				Mix 1 teaspoon of the sap of the plant to 1 coffee cup of water and let to the chickens drink the preparation every morning for 2-3 days	Cholera	Chicken	
*Asparagus africanus* Lam.	Asparagaceae	Buticho^c^	S	Bark of *Olea eurepea* sub spp. *Cuspidate*+root of *Asparagus africanus* Lam.+non bursting seed of “*Ecalyptus camaldunesis* or *Ecalyptus globules*” should be dried, pounded and mixed together then add 1 cork of the mixture to 1 L of water, given orally 1 L/day for 2 or 3 days	Blackleg, pneumonia, and bloat	Cattle	AN7
*Basella alba* L.	Basellaceae	Dore^d^	H	The fresh Vegetative part of *Basella alba* L.+the flower of *Acmella caulirhiza* Del. are pounded, mixed together, and squeezed given 3 times/day (tid) until recovering:		Cattle, sheep, goat, and equine	AN40
				Orally	Bloat		
				Through eye	Eye problem		
				Topically	wound		
*Brucea antidysenterica* JF. Mill.	Simaroubaceae	Hatawo^c^	T	Add 1 L of water to the ground fresh seed given orally once per day for 3 days	Mastitis	Cattle	AN11
*Camellia sinesis*	Theaceae	Shayi^c^	S	After drying and crushing the fresh leaves of *Camellia sinesis* and *Nicotiana tabacum* L. mix with water to make paste then apply topically once	External parasites	Cattle goat and sheep	AN54
*Calpurnia aurea* (Ait.) Benth.	Fabaceae	Chekkata^b^	S	Add about one cup of water to the ground fresh leaf and given orally and topically once/day for 2 days: 100 ml - cattle 50 ml - goat and sheep	Internal and external parasite	Cattle goat and sheep	AN6
*Cardamine hirsuta* L.	Brassicaceae	Arabelada^b^	H	Two teaspoonful fresh ground root add to half cup of water and given half cup through nose for 2 days, daily	3 days sickness	Cattle	AN42
*Centella asiatica* (L.) Urban	Apiaceae	Not mentione^d^	H	pound the fresh leaf, mix with little water to prepare the paste then it is applied topically once	Itching	Sheep	AN30
*Chenopodium ambrosioides* L.	Chenopodiaceae	Not mentione^d^	H	After grinding the fresh leaf, mix with water to prepare (liquid) 1 L then it is given orally once	Mastitis	Cattle	AN27
*Clematis hirsuta* Perro and Guill	Ranunculaceae	Gale^b^	C	Pound the fresh leaf to make paste and apply (cover) on the wound	wound	All animals	AN1
*Cleome gynandra* L.	Capparidaceae	Ononu^b^	H	Five spoonful fresh ground leaf and vegetative parts add to 1 cup of water and given 1 cup through nose for 3 days, daily	Hepatitis	Cattle	AN23
*Comiphora erythrea* (Ehrenb.) Engl.	Burseraceae	Hagarsu^b^	T	Mix the sap of the plant with water and applied for 3 days, daily	External parasites	Ruminants	AN39
				Collect the sap of the plant and apply to the parts of the body (muscle) having get foreign materials inserted	To remove the foreign materials	All animals	
*Croton macrostachyus* Del.	Euphorbiaceae	Masencho^c^	T	The fresh leaves of *Croton macrostachyus*, *Trichlia* spp. and *Rhamnus prinodes* together crushed and mix with water and given orally 4 cup/day (morning and evening) for 34 days and apply topically	Diarrhea (dysentery), external parasite	Cattle	AN59
				By grinding about ½ kg of the fresh leaf including the vegetative part add to 1 L of water and given 1 L/day for adult and ½ L for calf for 2 days orally	bloat	cattle	
		Makkanisa^b^	T	Pound the fresh root, add water and filter then administered orally for 3 days (dog) and 7 days (other animals) and apply topically	Rabies, rectum prolapse, skin diseases (dermatophilosis, parasite)	Cattle, sheep, goat and equine	
*Dovyalis abyssinica* (A. Rich.) Warb.	Flacourtiaceae	Kerqicho^c^	T	The outer part of the fresh bark is removed and pounded then add about ½ kg to 1 L of water and administered orally 1 L/day for 2 days	Diarrhea	Cattle	AN41
*Ecalyptus camaldulensis* Dehnh.	Myrtaceae	Bbahr zaf^c^	T	Bark of Olea eurepea sub spp. Cuspidate+root of Asparagus africanus Lam.+ non bursting seed of “Ecalyptus camaldunesis” should be dried, pounded and mixed together then add 1 cork of the mixture to 1 L of water, administered orally 1 L/day for 2 or 3 days	Blackleg, pneumonia, and bloat	Cattle	AN55
*Ekebergia capensis* Sparrm.	Meliaceae	Olancho^c^	T	The outer part of the fresh bark is removed and crushed then add about ½ kg of the crushed bark to 1 liter of water and administered orally 1 L/day for 3 days	Constipation, general discomfort	cattle	AN13
*Erythrina brucei* Schwein.	Fabaceae	Welanko^c^	T	The fresh leaf is pounded and add 1 cup of water. It is given 1 cup per day orally for 3 days Applied topically	Internal parasite External parasite	Ruminants	AN12
*Euphorbia schizacantha* Pax	Euphorbiaceae	Harken^b^	H	Pound the fresh root then add enough water and given considerable amount orally for 4 days (morning and evening) Note: It is effective when mix with *Withania somnifera* (L.) Dunal	Anthrax	Cattle, sheep, goat and equine	AN44
*Ipomoea kituensis* Vatke	Convolvulaceae	Yemedir Embuay^b^	C	The dried root pounded, 1/2-1 teaspoonful powder is added to 1 tea cup water and administered orally and topical application; mouth - 3 days, daily and topical application 2 days interval for 3 days	Rectum prolapse	Cattle, sheep goat, and equine	AN24
*Iresine herbstii* Lindl.	Amaranthaceae	Abeba^d^	H	Pound the fresh leaf, mix with water to prepare 1 L (liquid) then it is administered orally once	Trypanosomiasis	Cattle	AN26
*Lapeirousia schimperi*	Iridaceae	Dhaqaabii^b^	H	The local healer chew the fresh bulb (root) and spit to the nose of the animal immediately after biting, and the dose is probably 1 teaspoon	Snake bite	Ruminants Equine	AN61
*Maesa lanceolata* Forssk.	Myrsinaceae	Abae^d^	S	The fresh leaves of *Maesa lanceolata Forssk* and *Nicotiana tabacum* L. are pounded together, add water and filter then 1 cc is administered through nose for 2 days	Leech	Cattle	AN28
*Momordica foetida* Schumach.	Cucurbitaceae	Berressa^b^	C	4 teaspoonfuls of the pounded fresh leaf add to 1 cup of water, 1 cup is administered orally for 8 days, 2-3 days interval	Babesiosis and/or Anaplasmosis	Cattle, sheep, and goat	AN21
*Momoridica boivinii*	Cucurbitaceae	Basu Bakula^c^	C	Fresh two fruits are pounded and add to 1 L of water and given 1 L orally, 3 days interval for 1 week	Pneumonia	Cattle and goat	AN36
*Myrtus communis* L.	Myrtaceae	Haddes^b^	T	pounded the fresh vegetative part of the leaf and after squeezing and filtering, add 4 teaspoonful of the filtration to 1 cup of water and administered orally 1 cup for 2 days, daily; morning and evening	Hepatitis	Ruminant and equine except camel	AN43
*Nicotiana tabacum* L.	Solanaceae	Tambo^b,c,d^	H	The fresh leaves of *Maesa lanceolata Forssk* and *Nicotiana tabacum* L. are pounded together, add water and filter then1cc is administered through nose for 2 days	Leech	Cattle	AN9
				After drying and grinding the leaves mix with water to make paste then apply topically once	External parasites	Cattle, sheep, goat and camel	
*Olea eurepea* subspp. *Cuspidate* (Wall. Ex G. Don) Cif.)	Oleaceae	Ajersa^c^	T	Bark of *Olea eurepea* sub spp. *Cuspidate*+root of *Asparagus africanus* Lam.+ non bursting seed of “*Ecalyptus camaldunesis*” should be dried, pounded and mixed together then add 1 cork of the mixture to 1 L of water, administered orally 1 L/day for 2 or 3 days	Blackleg, pneumonia, and bloat	Cattle	AN60
*Olinia rochetiana* A. Juss.	Oliniaceae	Nolle^c^	T	Add about 100 ml of water to the fresh ground leaf, administered orally once per day for 2 days (one informant) or Every 3 days for 8 days (different informant)	Mastitis, pneumonia, and other swellings or internal organs problems	Cattle, sheep, and goat	AN5
*Osyris quadripartita* Decn.	Santalaceae	Qorsa nyaataa^b^	T	Pound the fresh root and mix with water, filter and administered orally for 6-7 days, daily	Mastitis and poor mothering	Cattle, sheep, and goat	AN34
*Ozoroa insignis* Del.	Anacardiaceae	Gerri^b^	T	Dried bark and root of the plant is pounded then 2 teaspoonful powder added to 1cup of water, administered orally for 20 days, 2 days interval	Rabies	Ruminants Equine	AN22
*Ocimum lamifolium* (Roth)	Lamiaceae	Fafe^c^	H	The fresh whole part (especially vegetative part) is pounded, add about 20 g to 2 L of water, about 1 L is administered orally per day for 3 days	Diarrhea	Cattle	AN17
Phytolacca dodecandra L Herit.	Phytolaccaceae	Haranjicho^c^	S	the fresh leaf including the vegetative part is pounded and a small amount is added to 1 coffee cup of water then administered orally 1 coffee cup/day for 2 days	Dysentery and difficult urination	Cattle, sheep, and goat	AN15
*Ricinus communis* L.	Euphorbiaceae	Key qobo^c^	S	Pound about 50 g of fresh leaf and mix with 1 L of water then administered orally 1 L/day (every morning) for 2 days	Mastitis and poor mothering	Cow	AN53
*Rhamnus prinodes* L’ Herit	Rhamnaceae	Tado^c^	T	The fresh leaves of *Croton macrostachyus*, *Trichlia* spp. and *Rhamnus prinodes* together crushed and mix with water and administered orally 4 cup/day (Morning and evening) for 3-4 days and apply topically	Diarrhea (dysentery), external parasite	Cattle	AN49
*Solanum incanum* L.	Solanaceae	Hiddii^b^	H	After drying the root of both *Solanum incanum* L. and *Withania somnifera* (L.) Dunal plants, pounded, mix 1 teaspoonful from each plant and add water to make solution; 1 teaspoonful of the mixture is given as a drink for 3 days (animal) daily	Most diseases especially anthrax and three day sickness but wound	Cattle, sheep, goat and equine	AN2
				The local healer chew the fresh root and spit to the nose of the animal immediately after biting, and the dose is probably 1 teaspoon	Snake bite	Ruminant Equine	
*Solanum* spp.	Solanaceae	Roriko^d^	H	The fresh leaf and root are chewed by the local healer and spit to the mouth of the animal for 2 days	Mastitis and poor mothering	Cow	AN31
*Stephania abyssinica* (Dillon and A. Rich.) Walp.	Menispermaceae	Kalala^c^	C	The whole part; as it is, cut and put on the neck of the animal; until starting urination (there is fast recovery)	Blocking/difficult in urination	Horse	AN14
*Tragia brevipes* Pax	Euphorbiaceae	Lalesa^c^	C	The fresh whole part (especially vegetative part) is pounded, add about 20 g to 2-3 L of water, about 1 L is administered orally per day for 3 about 1 L is given per day for 23 days	Diarrhea	Cattle	AN37
*Trichilia* spp.	Meliaceae	Tewarako^c^	T	The fresh leaves of *Croton macrostachyus*, *Trichlia* spp. and *Rhamnus prinodes* together crushed and mix with water and administered orally 4 cup/day (morning and evening) for 3-4 days and apply topically	Diarrhea (dysentery), external parasite	Cattle	AN18
*Urera hypselodendron* (A. Rich.) Wedd.	Urticaceae	Hajija^d^	C	The fresh leaf is pounded and mix with water, and considerable amount is administered orally once	Retained placenta	Cattle and Sheep	AN25
*Vernonia amygdalina* Del	Asteraceae	Hecho^c^	T	Add about ¼ kg of the crushed fresh leaf to 3 L of water. It is administered orally for GIT problem about 1 L for 3 days, 2 days interval and applied topically for skin problem	Diarrhea and skin problem	Cattle	AN10
*Withania somnifera* (L.) Dunal	Solanaceae	Edigagga^b^	S	After drying the roots of both *Solanum incanum* L. and *Withania somnifera* (L.) Dunal plants, pounded, mix 1 teaspoonful from each plant and add water to make solution; 1 teaspoonful of the mixture is given as a drink for 3 days, daily	Most diseases especially anthrax and 3 day sickness but wound	Cattle, sheep, goat and equine	AN4
*Zaleya pentandra* (L.) Jeffrey	Aizoaceae	Araddoo^b^	H	After pounding the fresh root mix with clean water and administered through nose for 2 day	Nasal bot	Ovine	AN38

^a^Vouchers are stored in miniherbarium in the College of Agriculture and Natural Resources, Dilla University,

b Oromiffa,

c Sidamgna,

d Gedeoffa, GIT=Gastrointestinal tract, H=Herb; T=Tree, S=Shrub, C=Climber

**Table 2:** Growth habit of ethnoveterinary medicinal plants identified in the study area.

Habit	Frequency of response	Proportion (%)
Herb	18	36.7
Tree	17	34.7
Shrub	7	14.3
Climber	7	14.3
Total	49	100

### Methods and form of remedy preparation, dosages, and routes of administration

**Figure-2:**
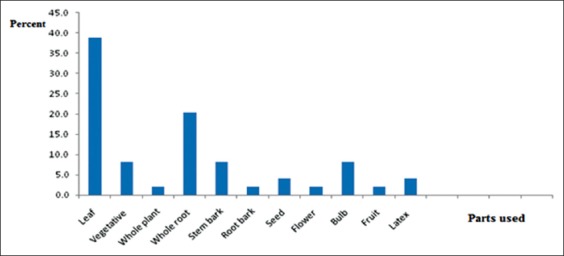
Plant parts used by the traditional healers for remedy preparation in the study areas.

About 63.3% ethnoveterinary medications were reported to comprise remedial parts of a single medicinal plant while 36.7% were prepared using formulations from two or more medicinal plant species either similar or different parts of the plants. Freshly harvested plant parts were the dominant ones (85.7%) used in remedy preparation whereas the remaining 14.3% of remedies were reported to be prepared from dried parts of medicinal plant species. The most commonly used plant parts for remedy preparations were leaves (38.8%), followed by whole roots (20.4%) (Figure-2).

Various methods of ethnoveterinary medicinal plants preparations were documented in this study based on type and form of animal ailments being treated. Pounding the remedial part and mixing it with water at room temperature was found to be the major method of remedy preparation (78.2%), followed by crushing and squeezing or chewing the remedial part of the ethnoveterinary medicinal plant without the use of water (12.7%). The remaining remedial methods preparations were collection of latex from the plants with or without the use of water (7.3%), and tie or put on (1.8%) the plant (climber) as it is on the neck of the animal. The most frequently used forms of preparation was liquid form (90.9%) and administered through mouth, nose, and eye, and applied topically followed by paste and latex (7.3%) and tie or put on (1.8%). However, two or more methods of remedy preparations and formulations were observed from a single plant depending on the type of disease to be treated (Table-1).

During preparation and dosage estimation of the local medicines, healers used various units of measurements; numbers (e.g., for seeds, fruits, bulbs, and flowers), spoon (e.g., for paste and powdered plant parts), and cups (e.g., for water during preparation and liquid form of the prepared medicine). However, no strictly standardized doses of herbal preparations as known for modern veterinary medicine were reported by traditional healers for any of the preparations used to treat livestock ailments in the present study. The dosage regime is generally dependent on the age and species of the animal. A single plant was found to be administered in different routes depending up on the preparation and type of the disease needed to be treated. Oral administration was found to be as the most frequently (63.3%) utilized route of administration followed by topical application (16.7%) (Table-3).

**Table-3:** Routes of administration of the ethnoveterinary medicinal plants identified in the study area.

Route of administration	Frequency of response	Proportion (%)
Oral	38	63.3
Topical	10	16.7
Nasal	8	13.3
Ocular	3	5.0
Tie on	1	1.7
Total	60	100

### Animal ailments and preference of ethnoveterinary medicinal plants

A total of 26 animal ailments were found in the area to be treated by a variety of medicinal plants (Table-1). The category: External parasite and skin problem have the highest ICF (0.68) followed by diarrhea and dysentery with 0.65 ICF (Table-4). Preference ranking of five medicinal plants that were reported as an effective treatment for external parasite and skin problem, which is the most common disease in the study area was conducted after selecting 6 key informants. *Calpurnia aurea* (Ait.) Benth was the most preferred effective treatment against the external parasite and skin problem followed by *Commiphora erythraea* in the study districts (Table-5). Three plants namely, *C. aurea* (Ait.) Benth, *Croton macrostachyus*, and *Nicotiana tabacum* L. were reported as treatment of external parasite and skin problem in all the study areas having different cultures and languages.

**Table-4:** Informant consensus factor by categories of diseases in the study districts.

Category of disease	Number of plant species	Number of informant cited	ICF
External parasite and skin problem	10	29	0.68
Diarrhea and dysentery	9	24	0.65
Internal parasite, nasal bot and leech	8	20	0.63
Snake poisoning	4	9	0.63
Bloat and constipation	8	18	0.59
Mastitis and poor mothering	7	15	0.57
Pneumonia	4	7	0.50
Eye problem	3	5	0.50
Anthrax, black leg and 3-day sickness	8	14	0.46
Others	14	19	0.28

ICF=Informant consensus factor

**Table-5:** Preference ranking of ethnoveterinary medicinal plants used for treating external parasite and skin problem in the study districts.

List of medicinal plants	R_1_	R_2_	R_3_	R_4_	R_5_	R_6_	Total	Rank
*Commiphora erythraea*	5	4	3	3	3	5	23	2^nd^
*Calpurnia aurea* (Ait.) Benth	5	4	4	5	3	5	26	1^st^
*Croton macrostachyus* Del	3	4	2	4	3	1	17	3^rd^
*Nicotiana tabacum* L.	3	3	4	2	2	1	15	4^th^
*Erythrina brucei* Schwein.	1	2	1	2	3	2	11	5^th^

R=Represented respondents

## Discussion

In the present study, 93.5% of traditional healers responded that they acquired their knowledge from their parents or close relatives. Moreover, the local healers have a very high intention to keep their ethnoveterinary knowledge secrete and none of them was ready to transfer their knowledge either freely or on incentive bases to other people. In line with the present study, other studies have reported that the highest medicinal plant knowledge acquisition by the healers was from parents or close relatives and they have a very high intention to keep their traditional knowledge secrete [8,19].

Forty-nine plant specimens having ethnoveterinary medicinal value were botanically classified and distributed into 34 families. In this study, the highest number of plant species having ethnoveterinary medicinal value were found in *Euphorbiaceae, Fabaceae* and *Solanaceae*, with 4 plant species each and *Amaryllidaceae* (3 species). In agreement with this study, *Asteraceae*, *Cucurbitaceae*, *Euphorbiaceae*, *Fabaceae*, and *Solanaceae* have also been reported as dominant families in other studies [18-20]. Moreover, consistent with this study Mesfin *et al*. [8] have reported that herbs were the most harvested for the ethnoveterinary medicinal purpose. However, discordant to this study shrubs have been documented as the most important ethnoveterinary medicinal plants in the other part of the country [20].

The most commonly used plant parts for remedy preparations were leaves (38.8%), followed by whole roots (20.4%). Consistent with this study, leaf has been identified as the most frequently used plant part [18,19]; however, in contrast to this study, Lulekal *et al*. [20] have found that root is as the most used part in their studies. This difference could be as the pharmaceutical value and concentration of active ingredients in each plant varied depending on climatic and edaphic factors. People inhabiting different ecological zones use different plants and plant parts in their treatment arsenal [15].

Results also indicate pronounced preference of traditional healers in the study districts to make use of freshly harvested plant parts (85.7%) for remedy preparation over dried forms. Similar observations were reported [10,16,20] for other cultural groups living in Ethiopia. This could be attributed to the wide-spread traditional belief of attaining high efficacy from fresh remedies due to higher presence of active ingredients in the form of secondary metabolites in cases of fresh plant parts which community members rightly thought could be lost on drying.

About 63.3% ethnoveterinary medications were reported to comprise remedial parts of a single medicinal plant in the present study which is in agreement with the findings of studies conducted elsewhere in Ethiopia [26] and Pakistan [27]. However, 36.7% of the traditional medications were also prepared using formulations from two or more ethnoveterinary medicinal plant species either similar or different parts of the plants for treating livestock ailments may be attributed to the expected synergetic effect of combinations of parts and their bioactive ingredients to treat ailments. Giday *et al*. [28] have also reported the therapeutic efficacy of combinations of medicinal plant parts used in other peoples living in northwest Ethiopia for treating various ailments.

Pounding the remedial part and mixing it with water at room temperature was found to be the most common method of local drugs extraction (78.2%), which is in line as documented in other studies [19,28]. Oral (63.3%) route of administration is popular as in the finding of Tamiru *et al.*, [19] who reported oral as the leading route of administration used in western Ethiopia. It is also in agreement with the result of various ethnobotanical studies conducted elsewhere in Ethiopia [8,15,20,29] which indicates oral as the predominant route of administration used by the herbalists. A single plant was found to be prepared in different formulations and administered in different routes depending up on the type of the disease needed to be treated as reported by Tamiru *et al*. [19].

Even though healers used various units of measurements to estimate doses of local medicines such as numbers (e.g., for seeds, fruits, bulbs, and flowers), spoon (e.g., for paste and powdered plant parts), and cups (e.g., for water during preparation and liquid form of the prepared medicine), no strictly standardized doses of herbal preparations as known for modern veterinary medicine were reported by traditional healers for any of the preparations used to treat livestock ailments in the present study areas. Similar findings have been reported in other studies [17,20].

*C. aurea* (Ait.) Benth, *Commiphora erythraea, Nicotiana tabacum* L., *Croton macrostachyus* Del, and *Erythrina brucei* Schwein were the ethnoveterinary medicinal plants identified and were used to treat external parasite and skin problem which was the most prevalent animal health problem reported in the study area. *C. aurea* (Ait.) Benth, *Nicotiana tabacum* L. and *Croton macrostachyus* Del have been reported [17] as treatment of external parasite and skin problem in animals in Tigray region, northern Ethiopia. Gebrezgabiher *et al*. [18] also documented that *C. aurea* (Ait.) Benth is being frequently used as ethnoveterinary medicinal plants for treatment of external parasite and skin problem in animals in northern Ethiopia.

## Conclusion

The study suggests that there is a vast amount of indigenous knowledge on ethnoveterinary medicinal plant and this knowledge plays an important role for the treatment of different animal ailments in the study districts. The healers have a very high intention to keep their traditional knowledge secrete and none of them was ready to transfer their knowledge either freely or on incentive bases to other people; they need to convey their knowledge only to their selected scions after getting very old. The knowledge is passed from generation to generation in an oral manner. Without being properly documented this information it could easily be lost or distorted. Commonly reported plant species need to be tested for their antimicrobial activities *in vitro* and validated their active ingredients in order to recommend effective preparations and treatments to this community.

## Authors’ Contributions

GRE has planned and designed the study. All authors conducted the field work. GRE has analyzed the data, supervised all stages of the work including during botanical identification of the plants and has written the draft of this report. All authors read and approved the final manuscript.
